# Bio-adsorption of heavy metals from aqueous solution using the ZnO-modified date pits

**DOI:** 10.1038/s41598-023-50278-y

**Published:** 2023-12-20

**Authors:** Khalid Khazzal Hummadi, Lin Zhu, Songbo He

**Affiliations:** 1 Joint International Research Laboratory of Circular Carbon, Nanjing Tech University, Nanjing, 211816 People’s Republic of China; 2 College of Engineering, University of Baghdad, 47024 Aljadria Baghdad, Iraq; 3 CoRe Pro BV, 9722NJ Groningen, The Netherlands

**Keywords:** Environmental sciences, Chemical engineering

## Abstract

The bio-adsorption of heavy metals (including Cu^2+^, Ni^2+^, and Zn^2+^) in aqueous solution and also in an industry wastewater using the ZnO-modified date pits (MDP) as the bio-adsorbent are investigated. The fresh and used bio-adsorbents were characterized by FT-IR, SEM, BET, and XRD. The bio-adsorption parameters (including the pH of solution, the particle size of MDP, the shaking speed, the initial concentration of heavy metals, the dosing of MDP, the adsorption time, and the adsorption temperature) were screened and the data were used to optimize the bio-adsorption process and to study the bio-adsorption isotherms, kinetics, and thermodynamics. Two adsorption models (Langmuir isotherm model and Freundlich isotherm model) and three kinetic models (pseudo-first-order model, pseudo-second-order model, and intra-particle diffusion model) were applied to model the experimental data. Results show that the maximum adsorption amount of Cu^2+^, Ni^2+^, and Zn^2+^ on a complete monolayer of MDP are 82.4, 71.9, and 66.3 mg g^−1^, which are over 4 times of those of date pits-based bio-adsorbents reported in literature. The bio-adsorption of heavy metals on MDP is spontaneous and exothermic, and is regulated by chemical adsorption on the homogeneous and heterogeneous adsorption sites of MDP surface. This work demonstrates an effective modification protocol for improved bio-adsorption performance of the date pits-based bio-adsorbent, which is cheap and originally from a waste.

## Introduction

Heavy metals (e.g., Cu^2+^, Ni^2+^, and Zn^2+^) in the contaminated water are persistent toxins and may accumulate in living organisms, causing different diseases and disorders^1^. The main sources of heavy metals are, e.g., electronic and cables industry (Cu), nickel alloy production (Ni), and brass coating (Zn)^2^. Levels of heavy metals in wastewater vary with the sources and locations^3^, e.g., concentration of Zn is between 0.16–56.7 mg L^−1^^4^. According to World Health Organization—Guidelines for drinking-water quality, the permissible limits of heavy metal ions in drinking water are e.g., 1.5, 0.1, and 5 mg L^−1^ for Cu, Ni, and Zn^5^.Several techniques such as coagulation^6^, ion exchange^7^, membrane filtration^8^, and precipitation^9^, for the treatment of heavy metals in solution have been developed. However, they either show low removal efficiency or are expensive in particular for treating heavy metals with a low concentration (e.g., < 100 ppm)^10^. Alternatively, adsorption has been found superior to the above techniques for the removal of heavy metals in terms of flexibility of design, initial investment cost, ease of operation, and low maintenance cost^11^. A variety of adsorbents (e.g., charcoal^12^ and activated carbon^13^) have been widely studied in literature and applied in the polluted water treatment plants.

In the latest development, the bio-adsorption technology using bio-based materials as the adsorbents (bio-adsorbents) has emerged and has become significant in the field^14^, including the removal of heavy metals in solution^15^. Different from adsorption, biosorption applies biological materials as the adsorbents^16^. A few bio-adsorbents (e.g., raw orange peel^15^, modified sugarcane bagasse^17^, modified oak sawdust^18^, modified Lignin^19^, modified sunflower stalks^20^, peanut hull^21^, dehydrated wheat bran^22^, maize leaf^11^, hazelnut shells^23^, Pinus bark^24^, banana and orange peels^25^, modified sugar beet pulp^26^, modified corncob^27^, Schleichera oleosa bark^28^, and bottlebrush plant seeds^29^) show the adorability for a wide range of heavy metals. Lignocellulosic biomass is a type of low-cost and abundantly available bio-adsorbent^30^ and shows the promise for the removal of heavy metals^31^. One of the examples could be the work by Rowell et al., who studied the bio-adsorption of Cu^2+^, Ni^2+^, and Zn^2+^ from aqueous solution using several lignin-containing agricultural materials^32^.

Date pits (DP) is an interesting lignocellulosic biomass^33^, which is a by-product of food processing and Jam production^34^ and has a large annual production of e.g., 600,000 tons in 2019, 750,000 tons in 2020, and 900,000 tons in 2021 in Iraq^35^. The potential of DP as a bio-adsorbent has been demonstrated by many research groups for the adsorption of heavy metals such as Au^3+^^36^, Cd^2+^^33^, and Al^2+^^37^. Two examples for the adsorption of Cu^2+^, Zn^2+^, and Ni^2+^ (that are interested in this study) could be the early work by Al-Ghouti et al. (showing the adsorption capacities of 0.15 mmol g^−1^ DP without any pretreatment for the removal of Cu^2+^)^33^ and the recent work by the authors (showing the adsorption capacities of 0.21, 0.15, and 0.13 mmol g^−1^ Al-Zahdi Iraqi DP for the removal of Cu^2+^, Zn^2+^, and Ni^2+^)^38^. Nevertheless, these bio-adsorption capacities of DP are lower compared to those of the other types of bio-adsorbents reported in literature^39^. Therefore, improving the adsorption capacity of DP-based bio-adsorbent remains the research interest.

In literature, modification (e.g., by coating^40^) of the raw bio-adsorbent towards a more effective bio-adsorbent has been widely studied^19,41^. Coating with ZnO is of significant research interest considering that ZnO is a comparatively inexpensive and environmentally benign material, and contains functional groups (e.g., -OH) in an aqueous medium that renders the absorption of metal ions^42^. ZnO, such as ZnO nanoparticles^43^ and nanorods^44^, could be used alone for the adsorption of heavy metals (e.g., Cd^2+^, Cu^2+^, Ni^2+^, Pb^2+^, Zn^2+^, Cd^2+^, Hg^2+^, and As^3+^) from solution, however, is often used in combination with other adsorbents (e.g., mesoporous carbon^45^ and graphene oxide nanocomposites^46^) for an improved removal of heavy metals from solution.

Therefore, based on the above promising adsorption performance of ZnO-containing adsorbents and the DP-based bio-adsorbents, ZnO-modified DP (MDP) could be an alternative bio-adsorbent for the bio-adsorption of heavy metals, which to the best of our knowledge, has not been considered in literature. In this contribution, the Al-zahdi Iraqi Date pits (DP) were modified by loading ZnO followed by thermal treatment. The MDP bio-adsorbent was characterized in detail and the isotherms, kinetics, and thermodynamics of the bio-adsorption of heavy metals (Cu^2+^, Zn^2+^, and Ni^2+^) in solution were studied systematically, focusing on the surface properties of the bio-adsorbent and the consequent effect of bio-adsorption conditions (adsorbent concentration, adsorbent particle size, shaking speed, pH of the solution, initial concentration of heavy metals, adsorption time, and adsorption temperature) on the bio-adsorption performance. The present work shows the significance of the modification of date pits (DP)-based bio-adsorbent by loading of ZnO and the followed thermal treatment, which remarkably improved bio-adsorption efficiency for the removal of heavy metals (Cu^2+^, Ni^2+^, and Zn^2+^) in the solution compared to those in literature.

## Experimental

### Preparation of the bio-adsorbents

**Figure 1:**
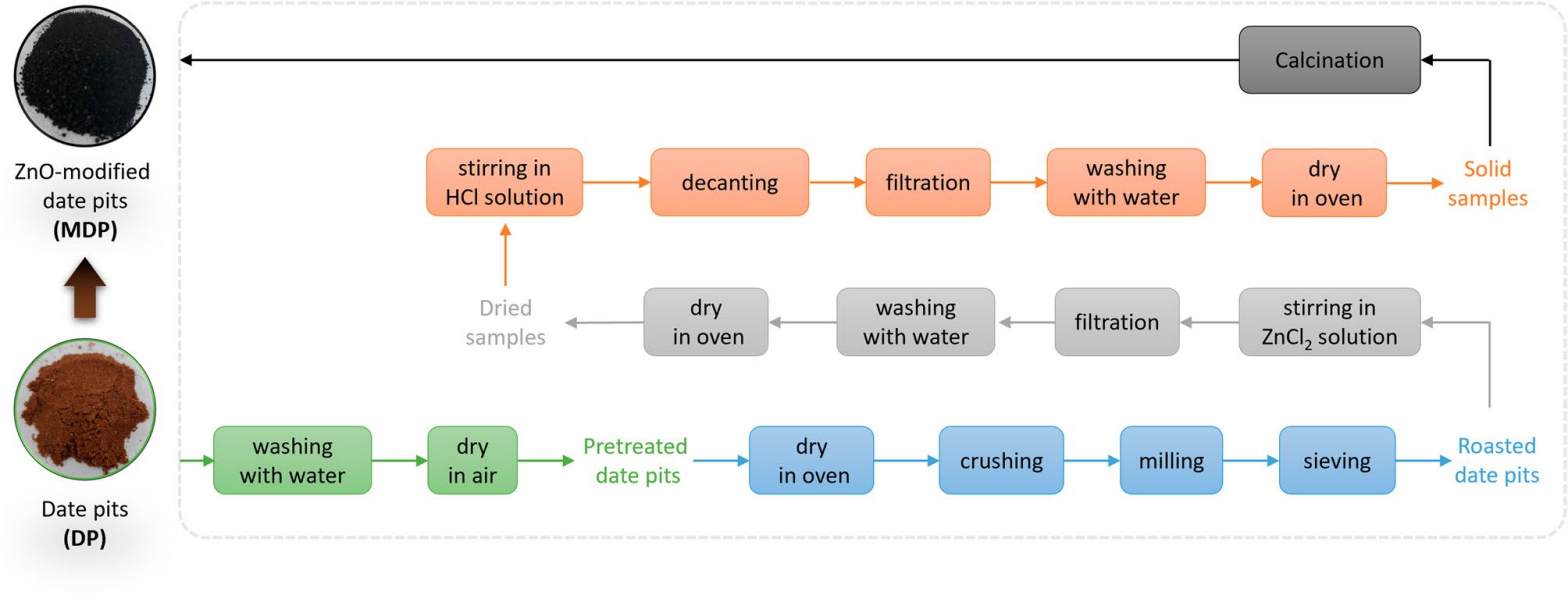
Photos of date pits (DP) and ZnO-modified date pits (MDP) (left) and the steps for the preparation of MDP from DP (right).

The steps for the preparation of ZnO-modified date pits (MDP) from date pits (DP) are shown in Fig. 1. Iraqi Zahedi date was bought from the local market in Baghdad. The obtained date pits (DP, Fig. 1) were cleaned and washed 3 times with deionized water and were dried in air for 7 days. The pretreated DP were further dried in an oven at 120 °C for 24 h, followed by crushing, milling, and sieving to obtain the roasted DP with particle sizes of 1500, 1000, 500, and 200 µm. The roasted DP (10 g) were then added to aqueous zinc chloride solution (2 M, 100 ml) in a 500-mL flask and the mixture was stirred (150 rpm) at 80 °C for 4 h, followed by filtration-washing for 5 cycles with deionized water and drying in an oven at 120 °C for 12 h. The dried samples were further added to aqueous HCl solution (0.1 M, 50 ml) and the mixture was stirred (50 rpm) for 2 h, followed by decanting, filtration, and washing the precipitate with deionized water for 5 times. After drying in an oven at 120 °C for 24 h, the solid samples were heated to 350 °C at a heating rate of 5 °C min^−1^ and were calcined for 4 h in a furnace. The obtained samples were denoted as modified date pits (MDP, Fig. 1) and stored in a desiccator. All procedures were conducted in accordance with the guidelines.

### Characterization of the bio-adsorbents

The fresh and spent MDP were characterized to study the characteristics of adsorbents for an understanding of the process and mechanisms involved in the bio-adsorption. Fourier transform infrared (FT-IR) spectra were obtained on an FT-IR spectrometer (Perkin Elmer Spectrum 2000). N_2_ physisorption was performed on a Surfer Gas Adsorption Porosimeter (Thermo Scientific). The specific surface area was determined using the Brunauer–Emmett–Teller (BET) method according to ASTM D1993. Scanning electron microscopy (SEM) was performed on a JSM-6090LV scanning electron microscope (JEOL). X-Ray Diffraction (XRD) patterns were obtained on a Magix Pro MPD X-ray diffractometer (Philips/Panalytical). The pH of the point of zero charge (pH_pzc_) was determined on a pH meter (inoLab 7110, WTW) according to the method described in Ref^47^.

### Bio-adsorption of heavy metals from aqueous solution and real wastewater

Bio-adsorption of heavy metals (including Cu^2+^, Ni^2+^, and Zn^2+^) from aqueous solution was performed in a batch system using MDP as the adsorbents. The metal salts namely copper sulfate pentahydrate (CuSO_4_·5H_2_O), cobalt(II) nitrate hexahydrate (Ni (NO_3_)_2_·6H_2_O), and zinc sulphate heptahydrate (ZnSO_4_·7H_2_O) were dissolved in 50 mL water in a 100-mL flask, followed by adding the adsorbents under shaking. The adsorption conditions were: pH of 2–8, adsorbent dosing of 0.4–6 g L^−1^_solution_, adsorbent particle size of 200–1500 µm, shaking speed of 150–400 rpm, adsorption time of 10–180 min, initial concentration of heavy metals of 10–90 mg L^−1^, and adsorption temperature of 25–55 °C. The liquid samples were taken at different adsorption time intervals and were analyzed on a GBC 932 atomic absorption spectrometer after the filtration using cellulose nitrate membrane with pore size of 0.45 µm.

After the bio-adsorption, the sludge (namely the spent MDP bio-adsorbent) was regenerated and reused to evaluate the reusability of the MDP. The spent MDP containing the adsorbed heavy metals was added to 14 ml aqueous solution containing 50 mM H_2_SO_4_. The slurry was stirred at a speed of 350 rpm and at a desorption temperature of 25 °C continuously for 48 h. After the filtration and washing with water, the MDP was reused for the bio-adsorption for 4 times.

In addition, a real wastewater, which was collected from an electroplating company in Baghdad and contained 21.6 mg L^−1^ of Cu^2+^, 13.9 mg L^−1^ of Ni^2+^, and 18.9 mg L^−1^ of Zn^2+^, was applied to evaluate the bio-adsorption performance.

The removal efficiencies of heavy metals (R, %) and the adsorption capacity of the bio-adsorbent (q_e_, mg g^−1^)^47,48^ were calculated using Eqs. (1)^48^ and (2)^49^, in which C_0_, C_t_, and C_e_ are the initial concentration, the concentration at adsorption time (t, min), and the equilibrium concentration of heavy metals in the aqueous solution (mg L^−1^), respectively; v is the volume of the solution (L); and w is the weight of the bio-adsorbent (g). All the experiments were triplicated and the averaged data are reported.

1 $$R=\frac{{C}_{0}-{C}_{t}}{{C}_{0}}\times 100$$

2 $${q}_{e}=\frac{\left({C}_{0}-{C}_{e}\right)\times v}{w}$$

## Results and discussion

### Characterization of the bio-adsorbents

**Figure 2:**
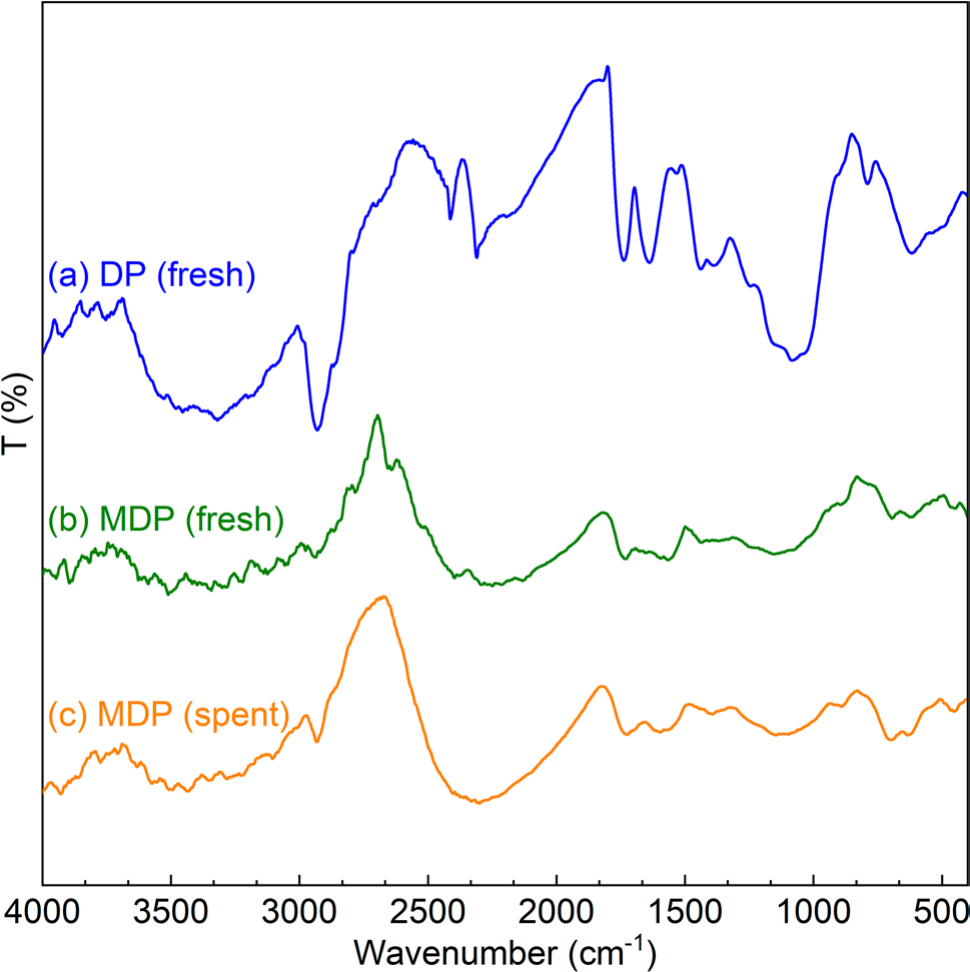
FT-IR spectra of (**a**) DP, (**b**) fresh MDP, and (**c**) spent MDP after simultaneous adsorption of heavy metals (at bio-adsorption conditions: adsorption temperature 25 °C, initial concentration of 90 mg L^−1^, MDP dosing of 2 g L^−1^_solution_, adsorption time of 180 min, particle size of 200 µm, shaking speed of 300 rpm, and pH of 5).

**Figure 3:**
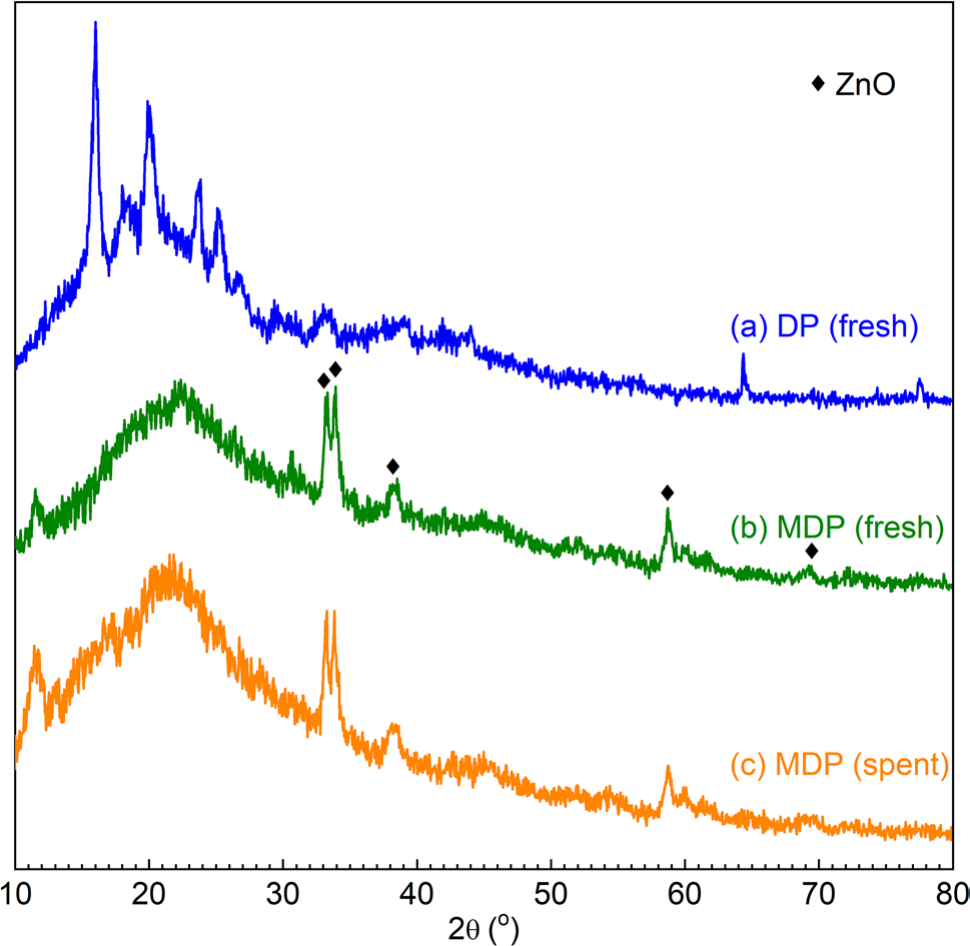
XRD patterns of (**a**) DP, (**b**) fresh MDP, and (**c**) spent MDP after simultaneous adsorption of heavy metals (at bio-adsorption conditions: adsorption temperature 25 °C, initial concentration of 90 mg L^−1^, MDP dosing of 2 g L^−1^_solution_, adsorption time of 180 min, particle size of 200 µm, shaking speed of 300 rpm, and pH of 5).

The specific surface area of Date pits (DP) is 82.4 m^2^ g^−1^. It consists of cellulose, hemicellulose, and lignin^50^. The FT-IR spectrum of DP (Fig. 2a) shows the typical adsorption bands of these biomass components, e.g., at 3500–3400 cm^−1^ (vibration of inter- or intramolecular hydrogen bonding (O–H) in cellulose^51^), 3000–2800 cm^−1^ (stretching of aliphatic C-H bonding^52^), 1745–1600 cm^−1^ (stretching of unconjugated C=O bonding in hemicellulose^53^), 1604.77 cm^−1^ (stretching of aromatic C=C bonding^53^), 1400–800 cm^−1^ (deformation of C-H bonding in lignin^54^), 1365 cm^−1^ (deformation of C-H bonding in cellulose and hemicellulose^52^), 1246 cm^−1^ (stretch of C–O bonding in lignin and xylan^52^), 1083.99 cm^−1^ (stretch of C–O bonding in hemicellulose^52^), and 869 cm^−1^ (stretch of C–O bonding in cellulose^52^). Besides, the XRD pattern of DP (Fig. 3a) also shows the representative diffraction peaks of crystalline cellulose (2θ = 16.2 and 23.9°^55^), amorphous hemicellulose (2θ between 30 and 50°^56^), and lignin (2θ = 64.4°^57^).

**Figure 4:**
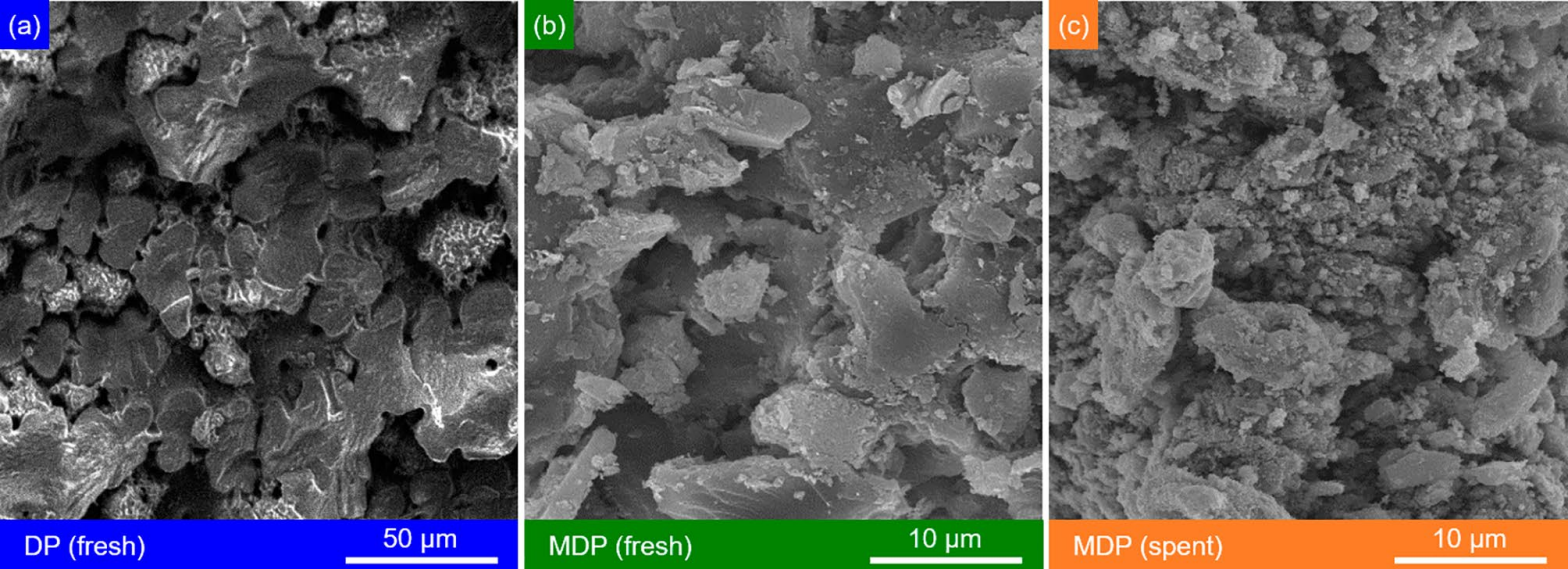
SEM images of (**a**) DP, (**b**) fresh MDP, and (**c**) spent MDP after simultaneous adsorption of heavy metals (at bio-adsorption conditions: adsorption temperature 25 °C, initial concentration of 90 mg L^−1^, MDP dosing of 2 g L^−1^_solution_, adsorption time of 180 min, particle size of 200 µm, shaking speed of 300 rpm, and pH of 5).

SEM image of DP is shown in Fig. 4a, indicating its rough surface with macropores^58^. The morphological characteristics of DP are changed after the incorporation of ZnO on DP (Fig. 4b), showing additional particles on the DP surface. EDX analysis of ZnO-modified date pits (MDP, Fig. S2) confirms the presence Zn and O, which are related to the ZnO particles. According to the EDX results, the ZnO content on MDP is about 35 wt.%. MDP contains ZnO particles with a particle size distribution of 0.1–1.2 μm (centered at 0.64 μm, Fig. S1, estimated by using the Digimizer program) and have a coarse (or unsmooth) surface (Fig. 4b). Compared to DP, MDP has a higher surface area of 195.89 m^2^ g^−1^, which is likely related to the loaded ZnO particles on the surface and is advantageous for the bio-adsorption (vide infra)^59^. It needs to be noted that MDP was treated at 350 °C, at which temperature the main components of DP could be partially converted, e.g., lignin starts to degrade at 200 °C, hemicellulose starts to decompose at 220 °C, and cellulose starts to depolymerize at 310 °C^60^. Since the thermal treatment temperature is higher than biomass torrefaction (e.g., 250–320 °C) but lower than the pyrolysis temperature (e.g., 450–600 °C), a bio-char-type material was likely obtained (Fig. 1). This is well reflected by the FT-IR spectrum of MDP (Fig. 2b, showing the disappearance and shifting of the adsorption bands for several functional groups on DP) and the XRD pattern of MDP (Fig. 3b, showing the disappearance of the diffraction peaks for the crystalline components in DP). The incorporation of ZnO onto DP is evidenced by the two characteristic adsorption bands of ZnO at 594 and 478 cm^−1^ (corresponding to the inorganic ZnO stretching^61^) in the FT-IR spectrum of MDP (Fig. 2b) and the diffraction peaks of ZnO at 2θ = 33.2°, 33.8°, 38.2°, 58.7°, and 69.1° (corresponding to the lattice planes of [100], [004], [101], [110], and [112], JCPDS card No. 00-036-1451) in the XRD pattern of MDP (Fig. 3b). ZnO crystal size was calculated using Scherrer equation (Eq. 3)^62^, in which K (= 0.9) is the Scherrer constant, λ (= 0.15406 nm) is the wavelength of the X-ray beam used, β is the full width at half maximum (FWHM) of the peak, and θ is the Bragg angle. The estimated ZnO crystal size is 7.0 nm, which is by far lower than the that of the particle size observed by SEM (vide supra). This indicates that the ZnO nanocrystals agglomerated on the surface of the date pits.

3 $$D=\frac{K\times \lambda }{\beta \times {\text{cos}}\theta }$$

After simultaneous bio-adsorption of heavy metals with an initial concentration of 90 mg L^−1^ at 25 °C and pH of 5 for 180 min, the morphological characteristics of the spent MDP (Fig. 4-) show distinct change to those of the MDP (Fig. 4-b), most likely related to deposition of the adsorbed heavy metal ions (including Cu^2+^, Ni^2+^, and Zn^2+^) on the spent MDP surface. ZnO is retained on the spent MDP according to the characteristic adsorption bands of ZnO in the FT-IR spectrum of the spent MDP (Fig. 2c) and diffraction peaks of ZnO in the XRD pattern of the spent MDP (Fig. 3c). The average ZnO particles on the spent MDP (Fig. 4c) is about 0.64 μm, comparable to that on the fresh MDP (vide supra). These characteristics indicate a good stability of MDP for the bio-adsorption of heavy metals.

### ***Bio-adsorption of Cu***^***2***+^***, Ni***^***2***+^***, and Zn***^***2***+^***using MDP***

#### Optimization of bio-adsorption parameters

Four bio-adsorption parameters, namely the pH of the solution, the particle size of the bio-adsorbent, MDP dosing, and the shaking speed, were screened to optimize the bio-adsorption performance. For this, the bio-adsorption experiments were performed for 180 min, assuming that the bio-adsorption reached equilibrium and a maximum removal of heavy metals from the solution was obtained.

##### Effect of pH of solution

**Figure 5:**
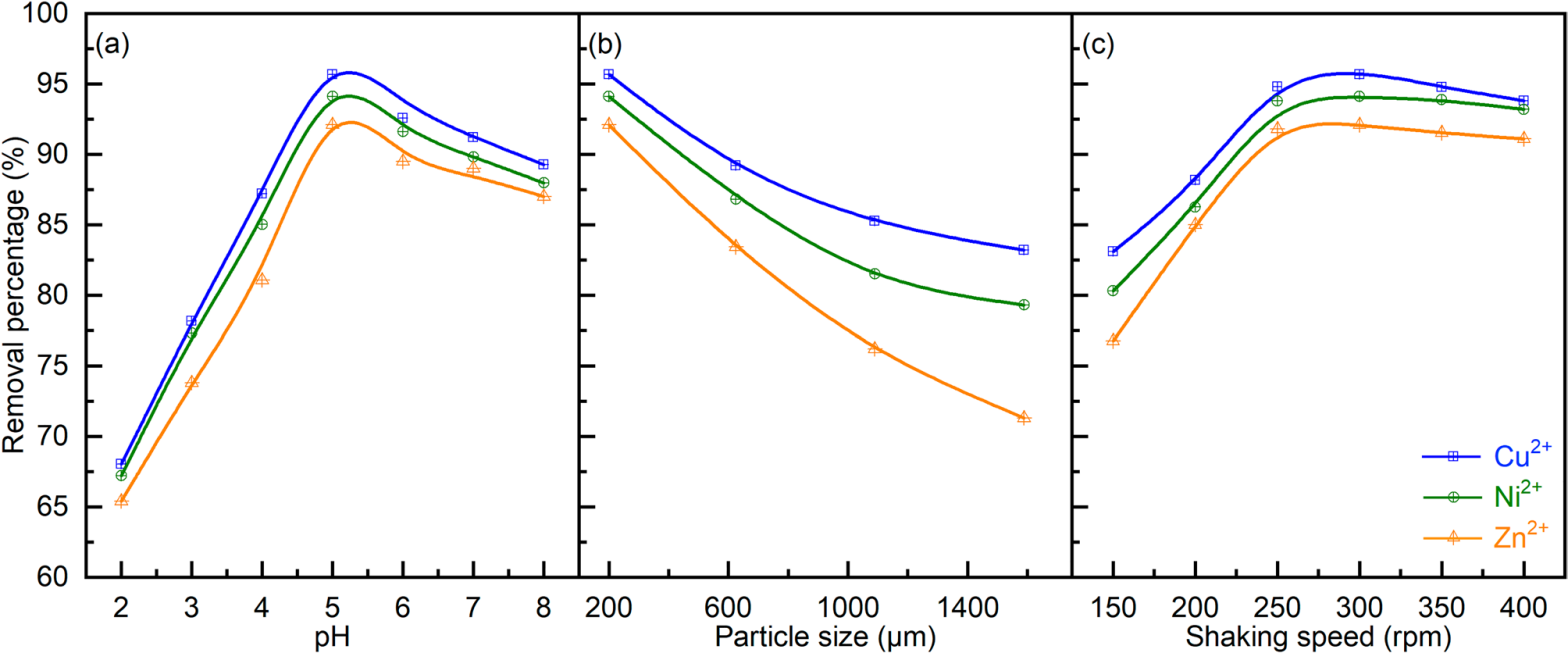
Effect of (**a**) pH, (**b**) particle size, and (**c**) shaking speed on the removal of heavy metals by the bio-adsorption on MDP. (Bio-adsorption conditions: adsorption temperature 25 °C, initial concentration of 50 mg L^−1^, MDP dosing of 2 g L^−1^_solution_, adsorption time of 180 min; (**a**) particle size of 200 µm and shaking speed of 300 rpm; (**b**) pH of 5 and shaking speed of 300 rpm; (**c**) pH of 5 and particle size of 200 µm.)

The amount of removed heavy metals (Cu^2+^, Ni^2+^, and Zn^2+^) by the bio-adsorption on MDP increases with the increase of the pH value from 2 to 5, followed by a decrease when the pH value is further increased, e.g., to 8 (Fig. 5a). This is likely due to that the varied pH of the solution of the adsorption system changes the solubilities of heavy metal ions, the concentrations of the counter ions on the adsorbent, and the degree of ionization of the adsorbent^63^, affecting the electrostatic interactions between positively charged heavy metal ions and negatively charged functional groups of the bio-adsorbent (such as −OH, −COOH, –O–, and −CO–NH–), which affects the adsorption capacity. At a low pH value, MDP has a positive surface charge. However, the bio-adsorbent is surrounded by hydronium ions (H_3_O^+^) with a high concentration in solution, leading to a repulsive force^22^ that hinders the access of the heavy metal ions to the adsorption sites of the bio-adsorbent. With the increase of pH, the surface of bio-adsorbent becomes less positive charge^64^ and may become neutral at a pH_pzc_ (point of zero charge) of *ca*. 6.1, which is estimated according to the experimental data shown in Fig. 5a. At a high pH value (e.g., > pH_pzc_), the heavy metal ions may react with the OH^−^ ions with a high concentration in solution^17^, which is detrimental for the bio-adsorption performance.

##### Effect of particle size of MDP

Removal of the three heavy metals by the bio-adsorption on MDP increases with the decrease of the bio-adsorbent particle size from 1500 to 200 μm (Fig. 5b). This could be related to the more amount of accessible adsorption sites on MDP, which were inside the big bio-adsorbent particles but are exposed to the external surface of the bio-adsorbent with a small particle size after crushing and grinding^38^. A further decreased particle size (< 200 μm) was not investigated, considering the significant difficulty of the separation of the fine bio-adsorbent particles from the solution.

##### Effect of shaking speed

Bio-adsorption of the three heavy metals on MDP increases with the increase of the shaking speed from 150 to 300 rpm (Fig. 5c), likely related to the enhanced external mass transfer of the metal ions from the solution to the active adsorption sites. Nevertheless, a further increasing the shaking speed from 300 to 400 rpm leads to a slight drop in the bio-adsorption performance. This might be attributed to the desorption of those heavy metals with a weak adsorption on the bio-adsorbent^65^.

##### Effect of MDP dose

**Figure 6:**
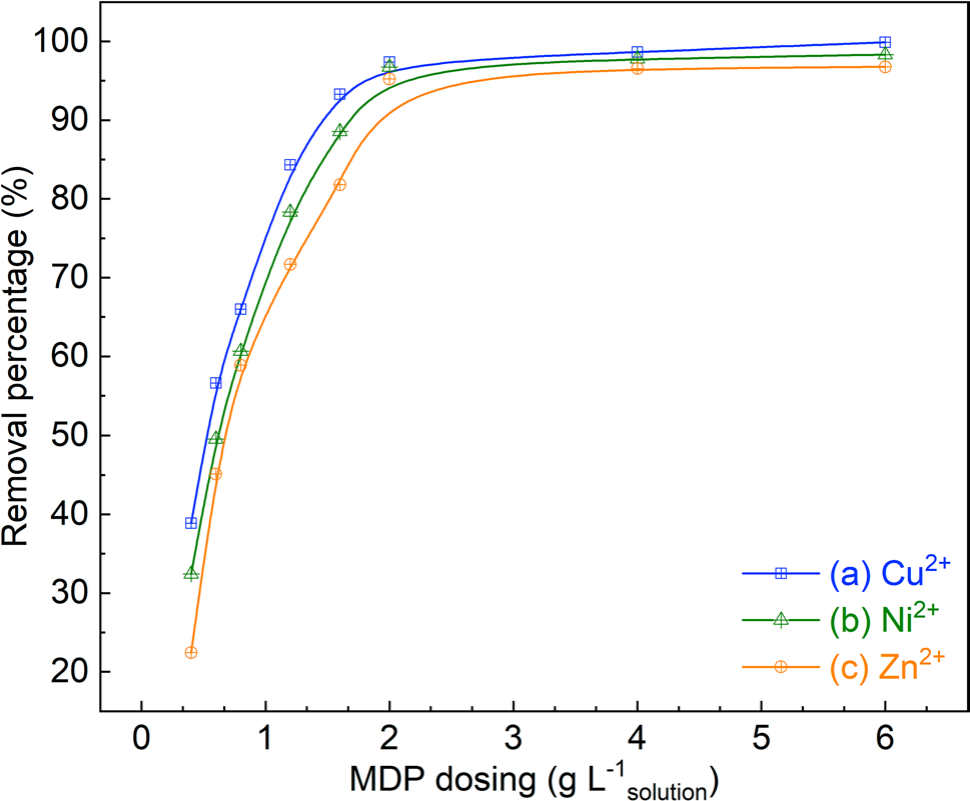
Effect of MDP dosing on the removal of heavy metals by the bio-adsorption on MDP. (Bio-adsorption conditions: adsorption temperature 25 °C, initial concentration of 90 mg L^−1^, MDP dosing of 0.4–6 g L^−1^_solution_, particle size of 200 µm, pH of 5, shaking speed of 300 rpm, and adsorption time of 60 min.)

It can be seen from Fig. 6 that the bio-adsorption efficiencies for the removal of Cu^2+^, Zn^2+^, and Ni^2+^ increase proportionately with the increase of MDP dose from 0.4 to 2 g L^−1^_solution_. This could be rationalized by the increased number of adsorption sites on the bio-adsorbents with a higher concentration in the solution. However, a further increasing the MDP dose, e.g., from 2 to 6 g L^−1^_solution_, does not show visible increase in the removal of the three metals (Fig. 6). On one hand, increasing the adsorbent concentration could cause the aggregation/agglomeration of adsorbent particles, leading to the dead points between the adsorbent particles that are not accessible for the adsorption^59^. On the other hand, the interaction/collision of the MDP particles with a high concentration in solution could lead to the desorption of the metal ions that weakly adsorbed on the adsorbent surface^25^. As such, an optimal MDP dose of 2 g L^−1^_solution_ is determined for the present study, under which conditions the removal percentages of 97.4%, 96.7%, and 90.2%, respectively, were obtained for the bio-adsorption of Cu^2+^, Ni^2+^, and Zn^2+^using MDP (Fig. 6).

##### Effect of the adsorption time

**Figure 7:**
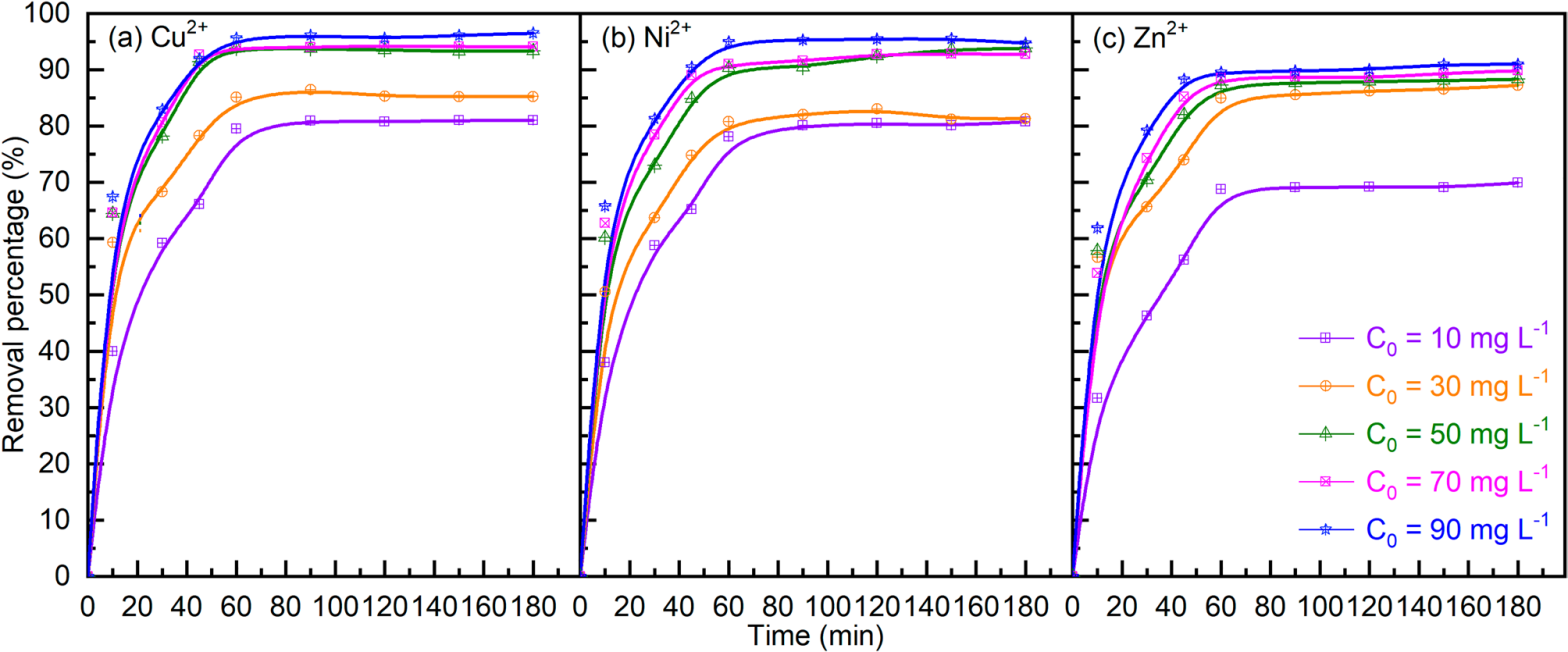
Effect of the initial concentration on the removal of Cu^2+^ (**a**), Ni^2+^ (**b**), and Zn^2+^ (**c**) by the bio-adsorption on MDP. (Bio-adsorption conditions: adsorption temperature 25 °C, MDP dosing of 2 g L^−1^_solution_, particle size of 200 µm, pH of 5, shaking speed of 300 rpm, and adsorption time of 10–180 min.)

In a batch bio-adsorption system, the removal percentage of heavy metals (Cu^2+^, Ni^2+^, and Zn^2+^) on MDP increases with the adsorption time (in the initial 10–60 min, after which plateau adsorption was reached, Fig. 7). This could be related to the fewer adsorption sites available on MDP and the decreased concentrations of heavy metals in solution with the adsorption time^66^. The latter is also evidenced by the higher efficiencies for removing heavy metals with higher concentrations shown in Fig. 7.

##### Effect of initial concentrations of heavy metals

The effect of initial concentrations of the three heavy metals (Cu^2+^, Ni^2+^, and Zn^2+^, Fig. 7) indicates that the removal percentage increases with increased initial concentration. This is very likely related to the increased driving force at a higher concentration’s gradient^67^.

##### Effect of adsorption temperature

**Figure 8:**
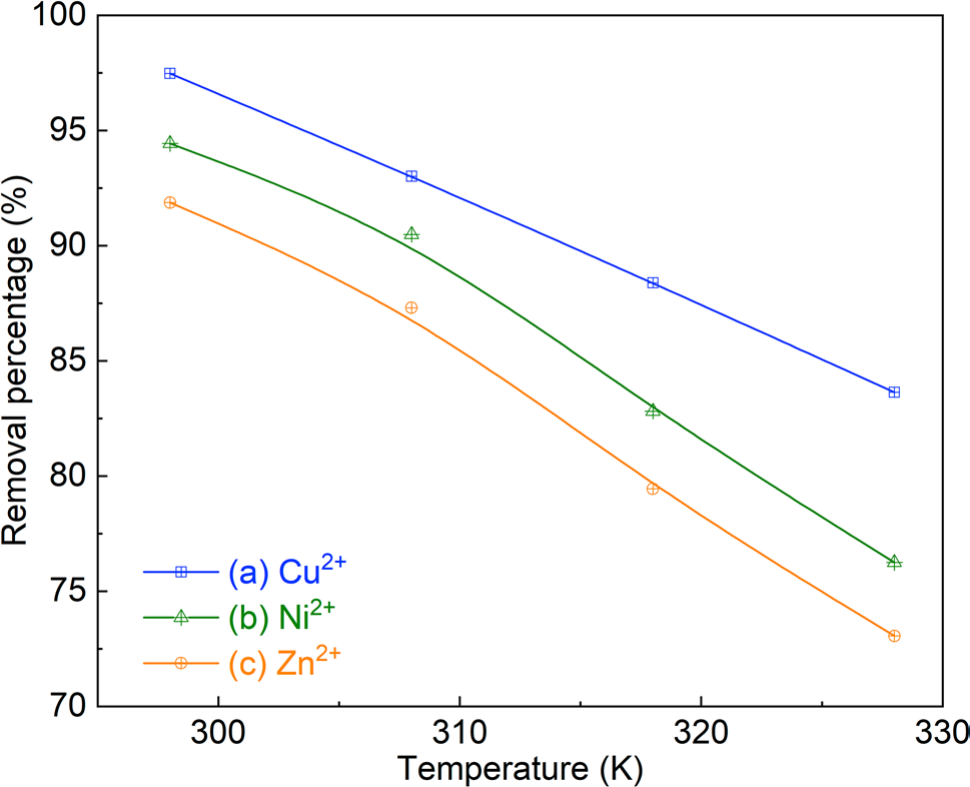
Effect of adsorption temperature on the removal of Cu^2+^ (**a**), Ni^2+^ (**b**), and Zn^2+^ (**c**) by the bio-adsorption on MDP. (Bio-adsorption conditions: initial concentration of 90 mg L^−1^, MDP dosing of 2 g L^−1^_solution_, particle size of 200 µm, pH of 5, shaking speed of 300 rpm, and adsorption time of 60 min.)

Increasing the bio-adsorption temperature (e.g., from 25 to 55 °C) has a negative effect on the removal of heavy metals (Fig. 8). One of the reasons could be due to the decreased viscosity of the aqueous solution at a higher temperature, resulting in an enhanced diffusion resistance to the bulk (external) and pore (internal) borders of the MDP particles^41^. Another reason might be related to the desorption of heavy metals from the adsorbent surface, which is favored at a higher temperature^68^.

#### Isotherm study

In literature, Langmuir^69^ and Freundlich^70^ adsorption models have been widely used for modelling the adsorption of heavy metals on various adsorbents^40^. In this study, the experimental data of the isothermal bio-adsorption equilibrium of the three heavy metals (Cu^2+^, Ni^2+^, and Zn^2+^) on MDP were also simulated using the above two models (Eqs. 4–6^71,72^) and the non-linear fitting curves are shown in Fig. S3. The constants of the two models and the correlation coefficients were calculated using nonlinear regression and are shown in Table 1.

**Table 1:** Parameters of the two isotherm models for the bio-adsorption of heavy metals on MDP.

	q_e exp_ (mg g^−1^)	Langmuir isotherm model					Freundlich isotherm model			
		K_Lan_	R_Lan_	q_m_ (mg g^−1^)	SSR^#^	R^2^	K_F_	n	SSR^#^	R^2^
Cu^2+^	77.5	0.359	0.03	82.4	28.6	0.964	28	3.37	8.28	0.980
Ni^2+^	68.2	0.279	0.03	71.9	25.2	0.939	23	3.19	2.09	0.940
Zn^2+^	50.5	0.194	0.04	66.3	20.5	0.878	17	3.00	1.08	0.910

^#^SSR = ∑(q_e cal_ − q_e exp_)^2^/N. SSR (Sum of squares due to regression) is the sum of the differences between the predicted value and the experimental data divided by N (total number of experiments).

4 $${q}_{e}=\frac{{q}_{m}\times {K}_{Lan}\times {C}_{e}}{1+{K}_{Lan}\times {C}_{e}}$$

5 $${q}_{e}={K}_{F}\times {C}_{e}^\frac{1}{n}$$

6 $${R}_{Lan}=\frac{1}{1+{K}_{Lan}\times {C}_{0}}$$

The fitting curves (Fig. S3) and parameters (R^2^ and SSR, Table 1) indicate that the bio-adsorption of Cu^2+^, Ni^2+^, and Zn^2+^ on MDP adjust better to the Freundlich isotherm model compared to the Langmuir isotherm model. This suggests^73^ that these heavy metals were adsorbed on the multi-layer and heterogeneous adsorption sites on the MDP surface. The maximum equilibrium adsorption amounts of Cu^2+^, Ni^2+^, and Zn^2+^ on a complete monolayer of MDP (q_m_) are 82.4, 71.9, and 66.3 mg g^−1^, respectively, according to the Langmuir isotherm model (Table 1). These q_m_ values are considerably higher than those reported in literature for the bio-adsorption of heavy metals on the date pits (DP)-based bio-adsorbents (Table 2,^38,74,75^) and also those for the roasted DP (without modification of ZnO, Tables 2), likely attributed to the modified characteristics of the DP upon loading of ZnO and the followed thermal treatment (vide infra).

**Table 2:** The adsorption capacity of heavy metals on the date pits (DP)-based bio-adsorbent.

Adsorbate	Adsorbent	Solution^d^	q_m_ (mg g^−1^)^e^	Reference
Cu^2+^	Raw DP	Single metal	31.3	Bouhamed et al.^74^
	Activated DP	Multi metals	18.7	Bouhamed et al.^75^
	Heated DP^a^	Multi metals	13.4	Hummadi^38^
	Roasted DP^b^	Multi metals	18.2	Present work
	MDP^c^	Multi metals	82.4	Present work
Ni^2+^	Raw DP	Single metal	24.4	Bouhamed et al^74^
	Activated DP	Multi metals	16.1	Bouhamed et al.^75^
	Heated DP^a^	Multi metals	7.8	Hummadi^38^
	Roasted DP^b^	Multi metals	11.6	Present work
	MDP^c^	Multi metals	71.9	Present work
Zn^2+^	Activated DP	Single metal	21.3	Bouhamed et al.^74^
	Activated DP	Multi metals	12.2	Bouhamed et al.^75^
	Heated DP^a^	Multi metals	9.7	Hummadi^38^
	Roasted DP^b^	Multi metals	9.8	Present work
	MDP^c^	Multi metals	66.3	Present work

^a^Heated at 80 °C, ^b^roased at 120 °C, ^c^modified by loading of ZnO followed by calcination at 350 °C, ^e^multi metals contain Cu^2+^, Ni^2+^, and Zn^2+^, and ^e^according to Langmuir isotherm model. Adsorption conditions in the present work: pH of 5, temperature of 25 °C, adsorption time of time 1 h, adsorbent dosing of 2 g L^−1^_solution_; in Ref^38^: pH of 5, temperature of 25 °C, adsorption time of time 72 h, adsorbent dosing of 12 g L^−1^_solution_; in Ref^74^: pH of 6, temperature of 20 °C, adsorption time of time 2 h, adsorbent dosing of 2.5 g L^−1^_solution_; and in Ref^75^: pH of 5.5, temperature of 20 °C, adsorption time of time 8 h, adsorbent dosing of 2 g L^−1^_solution_.

#### Kinetic study

The experimental kinetic data (Sections "Effect of the adsorption time" and "Effect of initial concentrations of heavy metals") were modeled to study the bio-adsorption kinetics, considering the practical implementation of a bio-adsorption system and also aiming to obtain insights into the types and mechanisms of the bio-adsorption of heavy metals on MDP. Two kinetic models, namely pseudo-first-order (Eq. (7) ^76^) and pseudo-second-order (Eq. (8) ^77^) that were widely used to model the adsorption of inorganic and organic matters from aqueous solution in literature^78,79^, were applied in this study. The parameters and the regression coefficients were calculated using the nonlinear fitting algorithms in the MATLAB program and are shown in Table 3. The good R^2^ values for the pseudo-second-order model indicate that pseudo-second-order model fits the bio-adsorption kinetics better than pseudo-first-order model.

**Table 3:** Parameters of various kinetic models for the bio-adsorption of heavy metals on MDP.

	C_0_ (mg L^−1^)	q_e exp_ (mg g^−1^)	Pseudo-first order model			Pseudo-second order model			Intra-particle diffusion model		
			q_e cal_ (mg g^−1^)	k_1_ (min^−1^)	R^2^	k_2_ (g mg^−1^ min^−1^)	q_e cal_ (mg g^−1^)	R^2^	k_IP_ (mg g^−1^ min^−1/2^)	C (mg g^−1^)	R^2^
Cu^2+^	10	4.22	3.85	0.070	0.899	0.002	4.22	0.958	0.187	1.97	0.989
	30	15.79	13.55	0.072	0.904	0.006	15.70	0.959	0.37	9.90	0.999
	50	25.32	23.27	0.100	0.938	0.005	25.32	0.978	0.69	16.06	0.980
	70	33.99	31.77	0.155	0.952	0.007	33.82	0.978	0.89	23.70	0.999
	90	43.54	41.58	0.173	0.962	0.007	43.53	0.978	1.029	31.62	0.993
Ni^2+^	10	4.40	3.86	0.061	0.899	0.010	4.40	0.985	0.193	1.84	0.999
	30	12.43	11.54	0.135	0.942	0.015	12.43	0.948	0.392	7.74	0.988
	50	22.96	21.49	0.147	0.961	0.015	22.32	0.975	0.619	15.49	0.951
	70	32.45	30.87	0.166	0.961	0.009	32.41	0.981	0.889	22.59	0.989
	90	43.46	41.12	9.169	0.964	0.007	43.42	0.983	1.062	30.83	0.993
Zn^2+^	10	3.84	3.39	0.049	0.903	0.010	3.84	0.943	0.177	0.15	0.999
	30	13.88	12.20	0.129	0.93	0.013	13.22	0.969	0.448	7.88	0.991
	50	22.54	20.77	0.151	0.953	0.011	22.14	0.981	0.624	14.74	0.985
	70	31.66	39.30	0.147	0.957	0.007	31.23	0.985	0.866	20.93	0.972
	90	41.27	39.21	0.172	0.965	0.008	41.03	0.985	0.986	29.64	0.993

7 $${q}_{t}= {{\text{q}}}_{e} \times (1- {{\text{e}}}^{{k}_{1}t} )$$

8 $${q}_{t}={k}_{2}\times {{q}_{e}}^{2}\times \mathrm{ t}/(1+{k}_{2}\times {q}_{e}\times \mathrm{t })$$

**Figure 9:**
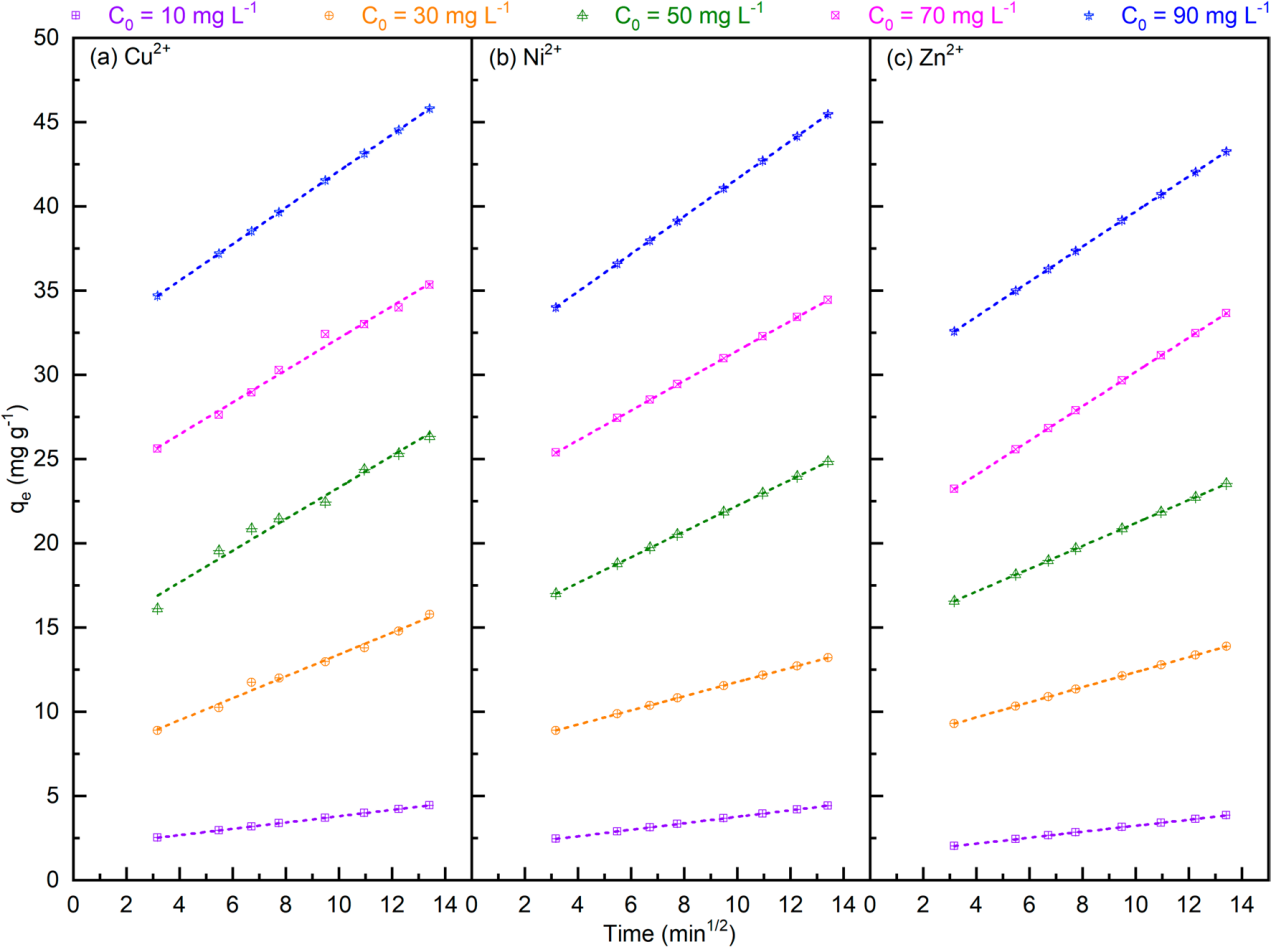
Intraparticle diffusion on the removal of Cu^2+^ (**a**), Ni^2+^ (**b**), and Zn^2+^ (**c**) by the bio-adsorption on MDP. (Bio-adsorption conditions: adsorption temperature 25 °C, MDP dosing of 2 g L^−1^_solution_, particle size of 200 µm, pH of 5, shaking speed of 300 rpm, and adsorption time of 10–180 min.)

In addition, the above experimental kinetic data were also analyzed using the intra-particle diffusion model (Eq. (9)^80^), which is often used to decide whether intra-particle diffusion is the rate-limiting step. It can be seen from Fig. 9 that the good linear fitting plots could be obtained, however, all the C values (in Eq. (9)) are greater than zero. These results suggest that the intra-particle diffusion mechanism is not dominant and very likely, there are also other mechanisms such as film, surface, or pore diffusion regulating the adsorption kinetics^81^.

9 $${q}_{e}={k}_{ip}\times {t}^{0.5}+C$$

#### Thermodynamic study

The experimental data of the effect of adsorption temperature (Section "Effect of adsorption temperature") were used to study the thermodynamics of the bio-adsorption of heavy metals on MDP. The thermodynamic parameters, namely Gibbs free energy change (ΔG , kJ mol^−1^), surface adsorption of entropy change (ΔS, kJ mol^−1^ K^−1^), and enthalpy change (ΔH, kJ mol^−1^) were calculated using Eqs. (10) ^40^ and (11)^82^ and the results are shown in Table 4. The three thermodynamic parameters (ΔG, ΔH, and ΔS) for the bio-adsorption at four temperatures (25–55 °C) are all negative values (Table 4), indicating that the adsorption of Cu^2+^, Ni^2+^, and Zn^2+^ on MDP is spontaneous (negative value of ΔG)^83^, exothermic (negative value of ΔH)^84^, and the randomness between the solid/liquid interfaces at the liquid–solid interface is decreased (negative value of ΔS)^85^.

**Table 4:** Thermodynamic analysis for the bio-adsorption of heavy metals on MDP.

	ΔG (KJ mol^−1^)				ΔH (KJ mol^−1^)	ΔS (J mol^−1^ K^−1^)	R^2^
	298 K	308 K	318 K	328 K			
Cu^2+^	− 9.054	− 6.628	− 5.366	− 4.448	− 54.181	− 152.73	0.968
Ni^2+^	− 7.014	− 5.768	− 4.156	− 3.180	− 46.152	− 131.46	0.991
Zn^2+^	− 6.006	− 5.432	− 3.574	− 3.003	− 38.502	− 108.66	0.951

10 $$\Delta G=-RT\times {\text{ln}}({k}_{d})$$

11 $${\text{ln}}\left({k}_{d}\right)=\frac{-\Delta H}{RT}+\frac{\Delta S}{R}$$

#### Bio-adsorption mechanisms

**Figure 10:**
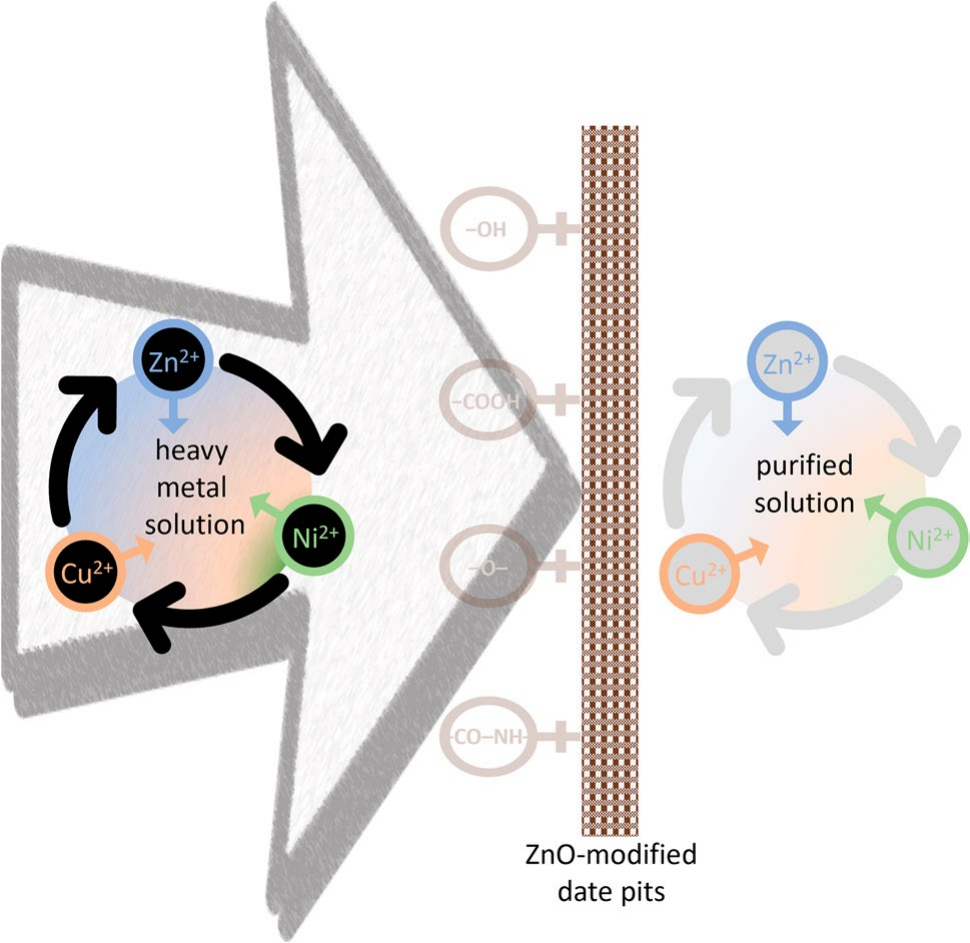
An illustration of the proposed Bio-adsorption mechanisms.

The adsorption process forms a layer of adsorbate (metal ions) on the surface of adsorbents. Bio-adsorbents, often have a porous structure with various cavities and surface sites (Fig. 4), on which the metal ions can be bonded. The improved pores and cavities on the MDP lead to an increased surface area for adsorption, promoting interaction between the metal ions with the bio-adsorbent^86^. Adsorption of the pollutant onto the adsorbent often includes three steps, namely transportation of the pollutant from aqueous solution to the adsorbent surface, adsorption onto the solid surface, and transport within the adsorbent particle. Kinetic study (Section "Kinetic study") indicates that both the intra-particle diffusion mechanism and the intra-particle diffusion mechanism are not dominant. The heavy metals have a vigorous affinity to the functional groups on the surfaces of the adsorbent^87^. On the surface of agricultural waste, functional groups such as −OH, −COOH, –O–, and −CO–NH–, react with heavy metal ions for their removal from aqueous solution^88^. Therefore, it is very likely that the electrostatic attractions between positively charged metal ions and negatively charged functional groups of bio-adsorbents promote the adsorption capacity^89^. Dispersion of ZnO on the DP surface improves the surface for electro-interaction. ZnO particles are generally found to have a positive value of the zeta potential^90^, which however, can be turned into a negative value because of the appearance of negatively charged centers, e.g., OH − groups on ZnO surface^91^. An illustration of the proposed bio-adsorption mechanisms is shown in Fig. 10.

#### Reuse of the spent MDP

**Figure 11:**
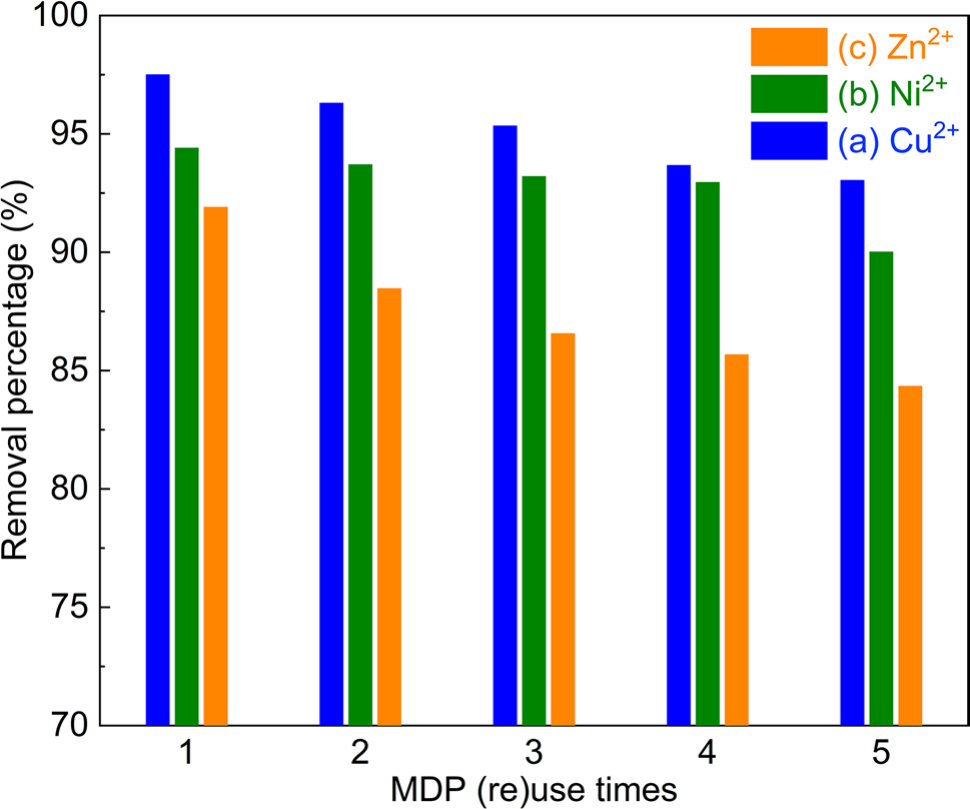
The removal percentages of Cu^2+^ (**a**), Ni^2+^ (**b**), and Zn^2+^ (**c**) by the bio-adsorption on the fresh and regenerated MDP. (Bio-adsorption conditions: initial concentration of 90 mg L^−1^, MDP dosing of 2 g L^−1^_solution_, particle size of 200 µm, pH of 5, shaking speed of 300 rpm, and adsorption time of 60 min.)

The reusability of the MDP bio-adsorbent was evaluated by recycling the regenerated MDP for additional 4 times. The used MDP, which was saturated with heavy metals during the bio-adsorption, was regenerated by following a desorption protocol described in Section "Bio-adsorption of heavy metals from aqueous solution and real wastewater". It can be seen from Fig. 11 that the removal percentage is decreased after each recycling. This indicates the irreversible deactivation of the MDP after the adsorption-regeneration cycle, of which the factors are under investigation and will be reported in due course.

### Bio-adsorption of a real wastewater using MDP

A real wastewater which was collected from a local electroplating company in Baghdad was applied to evaluate the bio-adsorption performance using the optimized adsorption conditions above, namely MDP particle size of 200 μm, MDP dosing of 2 g L^−1^_solution_, the shaking speed of 300 rpm, adsorption time of 1 h, and the adsorption temperature of 25 °C. The initial and final concentrations of the heavy metals before and after bio-adsorption process, the removal percentage, and the permissible limits are shown in Table 5. It can be seen that the removal efficiencies are higher than 90%, however, the concentration of the heavy metals in the treated water are still higher than the permissible limits^5^ for using as the drinking water. This is likely due to the presence of the other heavy metals (such as cadmium and lead, out of this study), which leads to an adsorption competition on the MDF surface. As such, the treated water after the bio-adsorption could only be used for non-drinking water, e.g., agriculture drain.

**Table 5:** Bio-adsorption of real wastewater using MDP.

Heavy metals	Initial concentration (mg L^−1^)	Final concentration (mg L^−1^)	Removal efficiency (%)	Permissible limits for drinking water (mg L^−1^)^5^
Cu^2+^	21.57	1.84	91.5	1.5
Ni^2+^	13.89	1.29	90.7	0.1
Zn^2+^	18.89	1.88	90.1	5.0

## Conclusion

The present work has shown the significantly improved bio-adsorption efficiency of the date pits (DP)-based bio-adsorbent after modification by loading of ZnO and the followed thermal treatment for the removal of heavy metals (Cu^2+^, Ni^2+^, and Zn^2+^) in the solution. This is rationalized by the changes in the characteristics (morphology, crystallinity, surface functional groups, and the specific surface area) of the ZnO-modified DP (MDP), compared to those of the raw date pits. At an optimized bio-adsorption condition (e.g., pH of 5, the particle size of 200 μm, the shaking speed of 300 rpm, initial concentration of 90 mg L^−1^, MDP dosing of 2 g L^−1^_solution_, adsorption time of 60 min, and the adsorption temperature of 25 °C), a high bio-adsorption efficiency of MDP for the removal of Cu^2+^ (97.4%), Ni^2+^ (96.7%), and Zn^2+^ (90.2%) in the solution was obtained. Irreversible deactivation was observed during the 5 cycles of adsorption-regeneration. Bio-adsorption of a real wastewater showed that the treated water can only be used as for non-drinking water. The isothermal analysis by the Langmuir isotherm model and the Freundlich isotherm model showed that both heterogeneous and homogeneous adsorption sites on MDP surface were involved for the bio-adsorption of heavy metals. According to the Langmuir isotherm model, the maximum bio-adsorption amount of the three heavy metals on MDP follows the order of Cu^2+^ (82.4 mg g^−1^) > Ni^2+^ (71.9 mg g^−1^) > Zn^2+^ (66.3 mg g^−1^). The kinetic study using the pseudo-first-order model, the pseudo-second-order model, and the intra-particle diffusion model suggested that the nature of kinetic adsorption is chemical and the intra-particle diffusion mechanism is not dominant, indicating the multiple bio-adsorption mechanisms. The thermodynamic parameters (ΔG, ΔH, and ΔS) pointed out that the bio-adsorption of heavy metals on MDP was spontaneous and exothermic, and the randomness between the solid/liquid interfaces at the liquid–solid interface is decreased. The modification protocol developed in this work is facile and could be a universal treatment of the bio-adsorbents for an improved bio-adsorption performance in terms of the purification of polluted water containing heavy metals.

### Supplementary Information

Supplementary Information.

## Data Availability

Original data and relevant materials can be provided by Dr. Khalid Khazzal Hummadi upon request.

## Abbreviations

C: Constant indicating the thickness of the boundary layer controlling the intra-particle diffusion, mg g^−^^1^
C_0_: Initial concentration, mg L^−^^1^
C_e_: Equilibrium concentration, mg L^−^^1^
C_t_: Concentration at time of t, mg L^−^^1^
K_F_: Freundlich constant, mg g^−^^1^
K_Lan_: Langmuir constant, L mg^−^^1^
k_d_: Equilibrium constant, k_d_ = q_e_/C_e_, L g^−^^1^
k_ip_: Rate constant of the intra-particle diffusion model, mg g^−^^1^ min^−^^1/2^
k_1_: Rate constant of the first-order kinetic model, min^−^^1^
k_2_: Rate constant of the second-order kinetic model, g mg^−^^1^ min^−^^1^
n: Adsorption intensity of the adsorbent, g L^−^^1^
q_e_: Equilibrium adsorption amount per unit mass of the adsorbent, mg g^−^^1^
q_e exp_: Equilibrium adsorption amount per unit mass of the adsorbent according to the experiment, mg g^−^^1^
q_e cal_: Equilibrium adsorption amount per unit mass of the adsorbent according to the model, mg g^−^^1^
q_m_: Maximum equilibrium adsorption amount on a complete monolayer of the adsorbent, mg g^−^^1^
q_t_: Adsorption amount per unit mass of the adsorbent at time of t, mg g^−^^1^
R: Universal gas constant, 8.314 × 10^–^^3^ kJ mol^−^^1^ K^−^^1^
R_Lan_: A dimensionless constant recognized as the separation factor
T: Solution temperature, K
t: Adsorption time, min
v: Volume of the solution, L
w: Weight of the bio-adsorbent, g
ΔG: Gibbs free energy change, kJ mol^−^^1^
ΔH: Surface adsorption of enthalpy change, kJ mol^−^^1^
ΔS: Surface adsorption of entropy change, kJ mol^−^^1^ K^−^^1^
