# Supplementary material for: Bio-adsorption of heavy metals from aqueous solution using the ZnO-modified date pits
Source: Sci Rep. 2023 Dec 20;13:22779. doi: 10.1038/s41598-023-50278-y (PMC10733537; doi:10.1038/s41598-023-50278-y)
Supplement: Supplementary file 1 — Supplementary Information. [file 41598_2023_50278_MOESM1_ESM.docx]

Supporting information

**Bio-adsorption of heavy metals from aqueous solution using the ZnO-modified date pits**

Khalid Khazzal Hummadi^a,b,*^, Lin Zhu^a^, and Songbo He^a,c,*^

*^a^Joint International Research Laboratory of Circular Carbon, Nanjing Tech University, Nanjing 211816, PR China*

*^b^College of Engineering, University of Baghdad, 47024, Aljadria, Baghdad, Iraq*

*^c^CoRe Pro BV, 9722NJ Groningen, The Netherlands*

*Corresponding authors. E-mails: dr.khalid.hummadi@coeng.uobghdad.edu.iq (K. Hummadi), songbohe@gmail.com (S. He).





**Figure S1** Particle size distribution of ZnO on fresh ZnO-modified date pits (MDP).


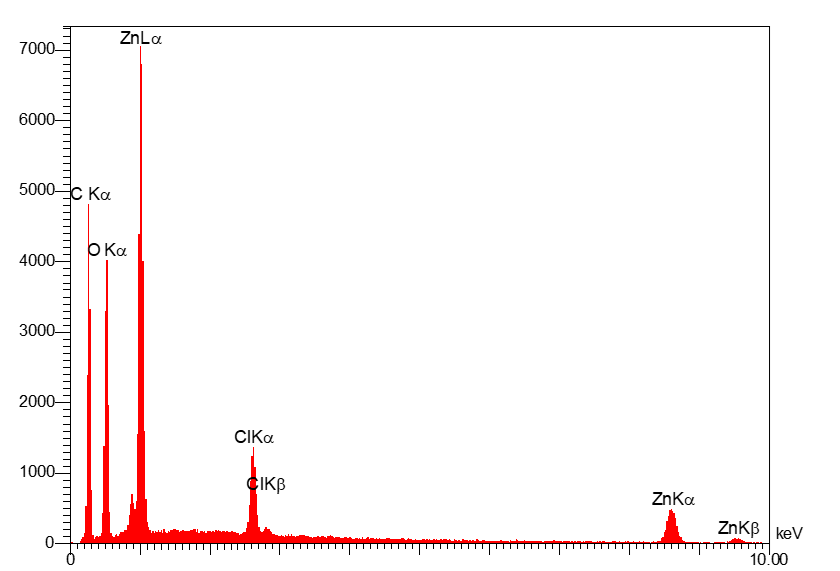


**Figure S2** Energy dispersive X-ray analysis (EDX) spectrum of the fresh ZnO-modified date pits (MDP).





**Figure S3** The equilibrium bio-adsorption of heavy metals on MDP and the corresponding non-linear fitting curves using the Langmuir and the Freundlich isotherm models. (Bio-adsorption conditions: adsorption temperature 25 °C, initial concentration of 90 mg L^-1^, MDP dosing of 0.4 - 6 g L^-1^_solution_, particle size of 200 µm, pH of 5, shaking speed of 300 rpm, and adsorption time of 60 min.)
